# Editorial: Medical food therapy for post-COVID conditions: mechanism and clinical study

**DOI:** 10.3389/fnut.2024.1409093

**Published:** 2024-04-16

**Authors:** Chester Yan Jie Ng, Ren-You Gan, Ning Wang, Linda L. D. Zhong

**Affiliations:** ^1^School of Biological Sciences, Nanyang Technological University, Singapore, Singapore; ^2^Singapore Institute of Food and Biotechnology Innovation (SIFBI), Agency for Science, Technology and Research (A^*^STAR), Singapore, Singapore; ^3^School of Chinese Medicine, Li Ka Shing Faculty of Medicine, The University of Hong Kong, Hong Kong, Hong Kong SAR, China

**Keywords:** post-COVID, medical food, COVID-19, alternative medicine (CAM), nutraceuticals

As the world navigates the ongoing repercussions of the COVID-19 pandemic, a prominent health concern is the emergence of “post-COVID conditions” (1). Post-COVID conditions encompass a variety of physical, social, and psychological challenges, which can significantly impact patients' health, wellbeing, and quality of life (2). While researchers have made strides in understanding the potential causes of these conditions, effective treatments strategies are still lacking. One potential approach is the use of medical food interventions, although the efficacy and mechanisms of medical food therapy in addressing post-COVID conditions require further investigation. Therefore, this Research Topic has gathered five insightful contributions from experts aiming to explore the management of post-COVID conditions through various research methodologies.

The first article by Tian et al. presented a meta-analysis evaluating the efficacy of probiotics in improving COVID-19 symptoms. Probiotics are a type of active microorganism beneficial to the host that changes the composition of a certain part of the host flora by colonization in the human body and have also been shown to reinforce immunity and counteract inflammation by restoring symbiosis within the intestinal microbiota (3). In the review, probiotics was found to have a significant impact on gut microbiota by regulating a variety of processes, including cell phenotypes, endocrine factors, and signaling pathways. The findings indicated that probiotics could enhance overall symptom improvement, reduce inflammation, and shorten hospital stays. Therefore, probiotics have great potential in managing gastrointestinal and respiratory symptoms through the gut-lung axis. Hence, one area of future research can aim to identify the specific strains of probiotics that are most effective in treating COVID-19 symptoms and explore their mechanisms of action. Another area of focus could also be on the large-scale development of probiotics into medical foods for wider consumption.

The second article by Li et al. revealed a causal association between the gut microbiome and COVID-19 using a Mendelian randomization analysis approach. The study highlighted the role of specific bacterial taxa in influencing COVID-19 severity and suggested that the gut-lung axis plays a significant role in the progression of the disease. It was observed that the genus *Alloprevotella* was causally associated with a higher COVID-19 severity risk and COVID-19 hospitalization causally increased the abundance of the phylum *Bacteroidetes*. Taken together with the findings from the first study by Tian et al., these findings suggest that therapies based on the gut microbiome have the potential to alleviate COVID-19. Hence, the gut microbiota may serve as biomarkers for predicting the prognosis of COVID-19, whereas microbiota-related interventions such as probiotics may alleviate symptoms and immune responses, as well as shorten the duration of symptoms in COVID-19 patients. Future studies can build upon these preliminary findings and explore the mechanisms by which the gut microbiome influences the prognosis of COVID-19 and development of post-COVID conditions.

The third article by Ng et al. delved into the use of Traditional Chinese Medicine (TCM) medicinal foods in the long-term treatment of post-COVID disorders. The study highlighted the importance of the TCM “Medicine and Food Homology” theory and suggested that medicinal foods could be a safe and effective therapy for managing symptoms associated with post-COVID conditions. In the case of chronic inflammatory diseases such as post-COVID conditions, TCM could potentially be a good treatment alternative due to its anti-inflammatory bioactivities (4). Furthermore, as a medicinal food, TCM herbs can be easily included into one's diet. An example is *Poria cocos* being transformed into food products such as Poria cake, which has been proved to alleviate digestive disorders (5). Therefore, further research can be conducted to elucidate the main bioactive compounds in TCM medicinal foods and their interactions with the human body, and to conduct larger scale randomized controlled trials to validate their efficacy in post-COVID management.

The fourth article by Duman and Karav reviewed the potential of bovine colostrum (BC) as a functional food in preventing COVID-19. BC is the initial milk an animal produces after giving birth and it contains numerous bioactive substances beneficial to human health (6). Hence, BC is utilized in the production of functional foods and medications to prevent a variety of ailments, including gastrointestinal and respiratory system disorders, all over the world. Given the positive effects of BC, the authors highlighted BC's potential in fostering a balanced immune response, which could be pivotal in managing COVID-19 severity. Therefore, future research can focus on finding specific BC components that play a role in COVID-19 treatment and prevention, as well as elucidating their mechanisms of action.

The last article by Tang et al. conducted a randomized, double-blind, placebo-controlled clinical trial aimed to evaluate the potential immunogenicity boosting effect of oral Huoxiang Suling Shuanghua Decoction (HSSD) after a third immunization with Sinovac's CoronaVac SARS-CoV-2 (CVS) inactivated vaccine. This study discovered that HSSD treatment significantly boosted serum anti-RBD IgG titer, decreased serum IL-6 levels, increased serum IgG, IgM, and C3 and C4 levels, and improved cellular immunity, as demonstrated by decreased balance deviations in lymphocyte subset distribution. Thus, this study demonstrated the potential of TCM immunomodulators in enhancing the immunological response to COVID-19 vaccinations. Future research directions could potentially include larger clinical trials including a more diverse population to improve the generalizability of the findings.

Collectively, medical foods can play an integral role in the management of post-COVID conditions. These contributions to the Research Topic provided a comprehensive review of the pathophysiology of post-COVID disorders, as well as prospective strategies for treatment. We hope the collected articles in this Research Topic will bring discussion and inspiration to relevant stakeholders about the use of medicinal food for the prevention, treatment, and management of post-COVID conditions. However, considering the recent emergence of post-COVID disorders, more research is needed to validate these findings at the biomolecular level and assess their efficacy in treatment. Therefore, we propose that future research should focus on understanding the mechanisms of action in post-COVID conditions, evaluating treatment efficacy in randomized controlled trials, and investigating the development of medical foods for the long-term treatment of COVID-19 and post-COVID conditions.

## Author contributions

CN: Conceptualization, Investigation, Writing – original draft, Writing – review & editing. R-YG: Conceptualization, Supervision, Validation, Writing – review & editing. NW: Conceptualization, Supervision, Validation, Writing – review & editing. LZ: Conceptualization, Supervision, Validation, Writing – review & editing.
